# New Proposal for Size and Size-Distribution Evaluation of Nanoparticles Synthesized via Ultrasonic Spray Pyrolysis Using Search Algorithm Based on Image-Processing Technique

**DOI:** 10.3390/ma13010038

**Published:** 2019-12-20

**Authors:** Elif Emil Kaya, Ozan Kaya, Gözde Alkan, Sebahattin Gürmen, Srecko Stopic, Bernd Friedrich

**Affiliations:** 1 Department of Metallurgical and Materials Eng., Istanbul Technical University, 34469 Istanbul, Turkey; emil@tau.edu.tr; 2 Department of Materials Science and Tech., Turkish–German University, 34820 Istanbul, Turkey; 3 Department of Mechatronics Eng., Istanbul Technical University, 34469 Istanbul, Turkey; kayaozan@itu.edu.tr; 4 IME Process Metallurgy and Metal Recycling, RWTH Aachen University, 52056 Aachen, Germany; galkan@metallurgie.rwth-aachen.de (G.A.); SStopic@metallurgie.rwth-aachen.de (S.S.); BFriedrich@metallurgie.rwth-aachen.de (B.F.)

**Keywords:** nanosilver, particle size, image processing, MATLAB, ultrasonic spray pyrolysis

## Abstract

Nanoparticle properties are correlated to their size, size distribution, and shape; it is essential to accurately measure these features in the field of nanoscience. In this study, silver nanoparticles (AgNPs) were synthesized with the ultrasonic-spray-pyrolysis (USP) method from a water solution of silver nitrate. The synthesized AgNPs were characterized by Dynamic Light Scattering (DLS) analysis and Scanning Electron Microscopy (SEM) to reveal their size and size distribution. A search algorithm based on an image-processing technique to obtain particle size and particle-size distribution from SEM micrographs is proposed. In order to obtain more quantitative information and data with respect to the morphology of particles synthesized under different process parameters, SEM micrographs with a nonhomogeneous background contrast were examined via image-processing techniques in MATLAB. Due to the inhomogeneous contrast of SEM micrographs, defining an overall threshold value was insufficient in the detection of whole nanoparticles. Thus, subimages were directly created according to the maximum and minimum particle size specified by the user to determine local threshold values. The obtained results were automatically combined to represent both particle dimension and location in the SEM micrographs. We confirmed that the results of our DLS analysis, theoretical calculation, and image-processing technique were correlated with our expected results.

## 1. Introduction

The unique chemical and physical properties of nanoparticles (NP) have led to their extensive use in various applications, including in the optical, electronic, magnetic, biomedical, and catalysis fields [1,2,3,4,5]. Silver NPs (AgNPs) have especially attracted considerable attention in diverse applications due to their superior properties, such as antimicrobial activity, and electrical and thermal conductivity [6,7,8,9]. Many methods have been employed for the synthesis of AgNPs: laser ablation, chemical reduction, solution combustion, and electrochemical and spray pyrolysis [10,11,12,13,14]. From these methods, the ultrasonic-spray-pyrolysis (USP) technique has been considered very promising since it enables the synthesis of pure, spherical, and fine nanoparticles as part of a one-step process. Moreover, this technique is capable of synthesizing metal/metal oxides with precisely controllable chemical composition, and particle size and morphology by manipulating process parameters such as solution concentration, temperature, gas-flow rate, and ultrasound frequency [15,16,17,18,19,20,21,22].

To obtain AgNPs with these desired properties, controlling and determining the size, size distribution, morphology, and composition of nanoparticles plays a critical role. For this purpose, analysis methods can be used for the detection of the properties of NPs. The mean diameter of NPs with a spherical shape synthesized by the USP technique can be theoretically calculated; however, particle-size distribution cannot be obtained by this calculation [21,22]. Particle size and size distribution can also be defined by Dynamic Light Scattering (DLS) analysis, but this requires extra analysis costs, and it remains difficult to accurately evaluate particle size and particle-size distribution due to the adhesion of nanoparticles. The measurement of particle size can be accomplished from Scanning Electron Microscopy (SEM) and Transmission Electron Microscopy (TEM) images. 

Compared to the DLS and electron-microscopy techniques, SEM and TEM provide more complete information about nanoparticle size and morphology. Since the manual counting and measurement of particles can be time-consuming, the functionality of a commercial software package was enhanced for the automatic measurement of particle size and size distribution from TEM and SEM micrographs. Applying a single threshold value, frequently used in commercial software, was insufficient to distinguish all particles in an image with nonhomogeneous background contrast. Measuring particle size and distribution is not feasible or easy via such commercial software due to the overlapping of particles during the sample-preparation stage for SEM and TEM analysis.

In the literature, several attempts were made to glean more information about the morphology and structure of materials via a computational approach using TEM and SEM images [23,24,25]. Ziel et al. obtained more information about the pore structure of membranes from SEM micrographs through an image-processing technique [25]. Rajeshwari et al. suggested a computational method for calculating particle diameters by measuring the distance between the two pixels selected at the particle boundaries [26]. Benitez et al. worked on an algorithm to achieve texture characterization and particle distribution in TEM images using a two-dimensional Hurst operator [27]. These methods, however, were insufficient to determine the exact diameter of nanoparticles due to the nonhomogeneous background contrast on SEM micrographs and overlapping nanoparticles.

In the present study, a new evaluation method was proposed for AgNPs synthesized through a USP technique in a vertical reactor. Despite SEM images having a nonhomogeneous background contrast, we propose a searching nanoparticle algorithm with an image-processing technique to reveal nanoparticle size and size distribution via MATLAB. Moreover, we conducted a comparative evaluation to determine particle size with an image-processing technique, DLS analysis, and theoretical calculation.

## 2. Materials and Methods 

Silver nanoparticles were synthesized with the USP method using 0.1, 0.2and 0.3 mol/L of aqueous silver nitrate solution (AgNO_3_, purity >99.9%). Experiments were conducted with an 8 L/min N_2_ flow rate over a 3 h period. There were 3 heating zones, set at 300, 800, and 300 °C, respectively, with the second zone being where the main thermal decomposition took place. The precursor was atomized by an atomizer with a frequency of 2.5 MHz and was carried into the preheated furnace through nitrogen gas flow. 

**Figure 1:**
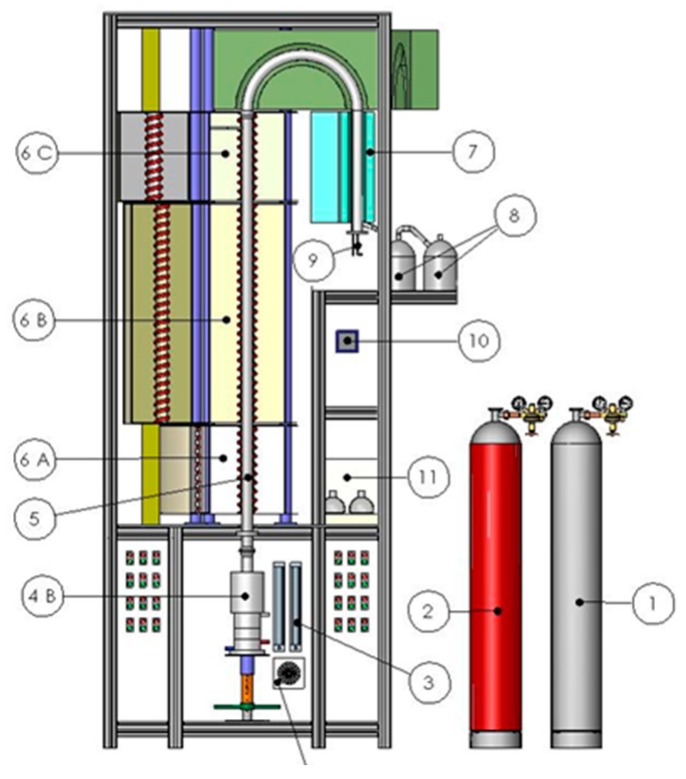
Experiment setup for ultrasonic-spray-pyrolysis (USP) synthesis of silver nanoparticles. (1) Bottle with hydrogen, (2) bottle with nitrogen, (3) flow meter, (4A) electronic unit, (4B) ultrasonic generator, (5) quartz tube, (6A) furnace (evaporation zone up to 300 °C), (6B) furnace (reaction zone up to 1100 °C), (6C) furnace (heat of up to 500 °C), (7) system for powder collection, (8) a bottle with water and alcohol, (9) connection with high-voltage device, (10) pressure indicator, (11) vacuum pump.

In the furnace, the reaction zone was 1.8 m wide and the diameter of the tube was 42 mm. The synthesized silver nanoparticles were collected in an electrostatic charged filter. The technical-scale experiment setup for synthesizing silver nanoparticles can be seen in Figure 1 [28,29].

The morphology and size of the silver nanoparticles were analyzed by SEM. We aimed to reveal the morphological features of the nanoparticles, such as their diameter, via image processing and a particle search algorithm. To detect the nanoparticles, threshold values were required to create a corresponding black and white image [30]. For this purpose, black and white views of SEM images were recreated using the threshold value [31]. Due to SEM micrographs having a nonhomogenous contrast background, using one threshold value was insufficient to detect all involved nanoparticles. Instead of using a single threshold value, an adaptive threshold value was applied, and images were divided into subimages to separate the nonhomogeneous background contrast area. Thus, the complexity of the threshold value of our SEM images was decreased. 

In line with this objective, subimages were created according to both maximum and minimum searching particle-size criteria defined by the user. This searching particle algorithm was designed to determine whether or not a nanoparticle was present in the subimages, and if the algorithm found one or more particles, they were then marked with their diameter and location. If our algorithm did not detect a particle, the searching particle method proceeded with the next subimage. Our searching particle algorithm can be seen in Algorithm 1.
**Algorithm 1** Searching particle Algorithm 1.1:Definition of min and max value for subimages2:Image reading3:**for** each defined value in size of image **do**4: Obtain grey code of subimage 5: Compute histogram of subimage6: Define threshold value7: Obtain black and white of subimage8: Search the particle9: **while** (detecting particle = 1) **do**10:   Measure particle size11:   Find location12:   Label particle13: **end while**14:**end for**

After being divided into subimages, the dimension of the subimages was scaled to extend each subimage [32], with each subimage becoming further detailed. Scaling is defined in Equation (1):

(1) $$\left[\begin{array}{c} x_{new}\\ y_{new} \end{array}\right]=\left[\begin{array}{cc} C_{1} & 0\\ 0 & C_{2} \end{array}\right]\left[\begin{array}{c} x\\ y \end{array}\right],$$

where *C*_1_ and *C*_2_ are scale factors for the *X* and *Y* axis, respectively.

For scaling subimages, new pixels were computed through a bilinear-interpolation method. Bilinear interpolation takes the average of 4 neighboring pixels to calculate a new pixel value [33]. Computing the value of the new pixel is defined in Equation (2):

(2) $$u\left(x\right)=\{\begin{array}{r} 0,\;\left|x\right|>1\\ 1-\left|x\right|,\;\left|x\right|<1 \end{array},$$

where *x* is the distance between the pixels and the interpolated pixel.

The Gaussian noise algorithm was applied to the image [34], and the histogram of each image was generated (the histogram method was preferrable for defining the threshold). Each threshold value was calculated by using the peak values of both dark and bright pixels on the histogram. With the Gaussian noise algorithm, smoothing the peaks of the histogram was achieved, and defining the threshold value for the black and white view of the subimages was simplified. The black and white view of the subimage was achieved using an adaptive threshold value. Furthermore, the stand-alone pixels in each boundary were erased via a kernel operation [35], with kernel operations illustrating the relation between pixels. In the case that the pixel had no relation to others, the values of the pixel were changed.

Two-dimensional convolution is given in Equation (3).

(3) $$Image_{new}\left(i_{1},j_{1}\right)=\sum_{n=1}^{3}\sum_{m=1}^{3}a\left(n,m\right)image\left(i_{1}-n,j_{1}-m\right)$$

**Figure 2:**
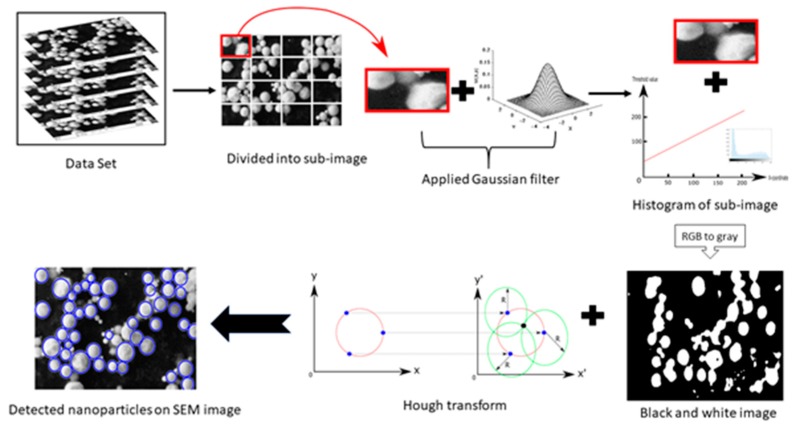
Flowchart of proposed method.

Nonaffiliated pixels were eliminated in the new image, and the diameter and location of the particles in our new black and white view were found. Since particles are known to have spherical shapes, the Hough transform method was utilized for labeling the nanoparticles [36]. The Hough transform draws new circles at the 3 smallest boundary points. Then, the center of the circle is calculated, with the junction point of new circles and diameter limits defined by the user. In the case where nanoparticles were shared by different subimages, both the location and diameter in the original SEM image were used to avoid confusion. The flowchart of the proposed method is shown in Figure 2.

## 3. Results and Discussion

Nanoparticle size and size distribution can be controlled by changing process parameters, but it is also important that they can be differentiated and accurately identified. In this study, the effect of precursor concentration in the range of 0.1, 0.2, and 0.3 mol/L on particle size and particle-size distribution was investigated by theoretical calculation, DLS analysis, and an image-processing technique via SEM micrographs. The theoretical mean particle diameter of silver nanoparticles was evaluated, assuming that all of the synthesized particles had a nearly spherical shape. The theoretical droplet size of silver nitrate was determined by Equation (4) [21,22]:

(4) $$D=0.34{\left(\frac{8.\pi .\gamma }{\rho .f^{2}}\right)}^{\frac{1}{3}}\;$$

where *D* is the mean droplet diameter, γ the surface tension of the solution, ρ the density of the solution, and f the ultrasound frequency.

The theoretical particle diameter of AgNPs was calculated using Equation (5):

(5) $$D_{p}=D{\left(\frac{C_{pre.}M_{Ag}}{M_{pre.\;}\rho_{Ag}}\right)}^{\frac{1}{3}}$$

where *Dp* is the mean particle diameter, *D* is the droplet diameter, *Cprec*. is the water concentration of the Ag(NO_3_) solution, and *ρ_Ag_* is the density of silver. 

We concluded that the increase in the precursor concentration from 0.1 to 0.3 mol/L caused an increase in the theoretical mean particle diameter of the synthesized silver, from 410 to 650 nm, which is consistent with previous findings [16]. The calculated values of the mean particle diameter are shown in Table 1. 

**Figure 3:**
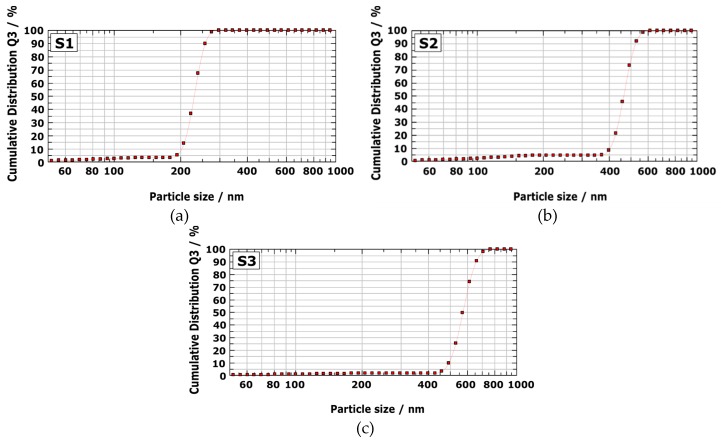
Dynamic Light Scattering (DLS) analyses of silver nanoparticles synthesized with varying solution concentrations. (**a**) S1; (**b**) S2; (**c**) S3.

The second technique we used was DLS analysis, which is widely known for determining particle size and particle-size distribution. DLS analyses of silver nanoparticles synthesized with varying solution concentrations at 800 °C are shown in Figure 3.

**Figure 4:**
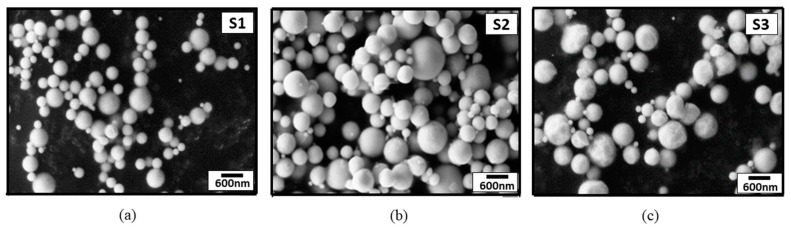
Scanning Electron Microscopy (SEM) micrographs of silver nanoparticles (AgNPs) synthesized by USP method. (**a**) S1; (**b**) S2; (**c**) S3.

It is evident from Figure 3 that there was narrow size distribution for AgNPs. The particle size of AgNPs using DLS analysis, as indicated in Figure 3a, showed a size range of 200–300 nm, with an average size of 258 nm. It is also clear that the prepared AgNPs, synthesized from 0.2 and 0.3 mol/L lay in the range of 400–600 nm and 450–700 nm, respectively. DLS analyses showed that the average size of AgNPs synthesized from 0.2 and 0.3 mol/L was around 526 and 653 nm, respectively. SEM micrographs of the AgNPs synthesized by USP from different solution concentrations can be seen in Figure 4.

Independent from the precursor solution concentration, typical spherical USP morphology was observed within all three samples, in agreement with findings in the literature [17,19,20]. SEM micrographs of AgNPs synthesized by the USP method were used to reveal this morphology.

**Figure 5:**
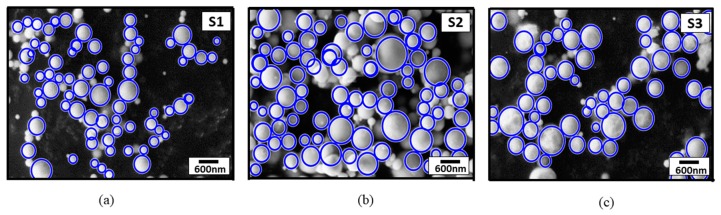
SEM micrographs of marked nanoparticles found via image-processing technique. (**a**) S1; (**b**) S2; (**c**) S3.

The morphological properties of nanoparticles were examined by a searching particle algorithm based on image-processing techniques. SEM images were randomly divided into subimages because of their nonhomogeneous background contrast. Thus, a smooth histogram was obtained for the definition of a threshold value for the black and white view of the SEM images. At this stage, adaptive threshold values were also used in the subimages due to each image’s nonhomogeneous contrast. The diameters of nanoparticles were computed via the Hough transform method. Moreover, minimum and maximum diameter limits were preliminarily defined to differentiate overlapping particles, and nanoparticle diameters on SEM images with a nonhomogeneous background contrast were roundly measured by a computational approach. The mean value and size distribution of the measured AgNPs were then calculated and plotted. The marked nanoparticles in the SEM micrographs, found via our image-processing technique, can be seen in Figure 5.

**Figure 6:**
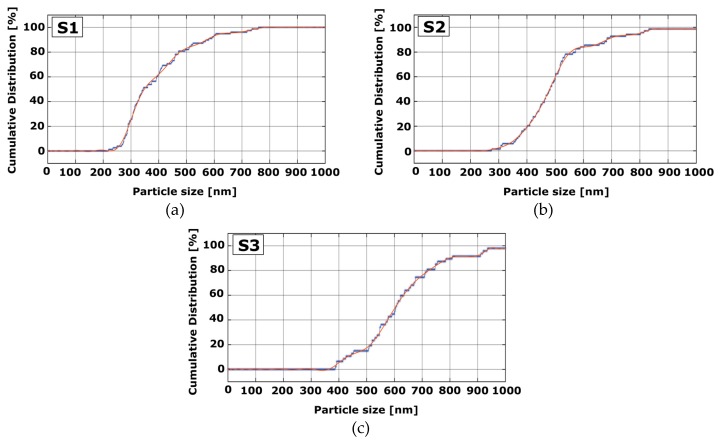
Plots of AgNP particle-size distribution obtained from SEM micrographs. (**a**) S1; (**b**) S2; (**c**) S3.

Graphs of the marked nanoparticles were plotted, with cumulative distribution represented by the y-axis, and nanoparticle size represented by the x-axis, as seen in Figure 6. The graphs of cumulative distribution versus nanoparticle size were drawn using polynomial fitting to achieve AgNP size distribution. The obtained discrete data of nanoparticle diameters were fitted to a 10th order polynomial. The polynomial order could be increased to further fit our graph’s curve. The obtained 10th order polynomial for these curves is as described in Equation (6):

(6) $$\begin{array}{ll} Con_{curve}\left(D\right)= & -3.0776D^{10}-2.88D^{9}+27.13D^{8}+18.77D^{7}-91.31D^{6}-35.95D^{5}\\ & +146.43D^{4}+3.52D^{3}-112.39D^{2}+67.56D+83.52 \end{array}$$

where *D* is the nanoparticle diameter, and the result of the polynomial is the percentage number of nanoparticle size. The results of our 10th order polynomial calculation for plots of cumulative distribution versus nanoparticle size are shown in Figure 6.

In Figure 6, the cumulative curve of AgNP particles whose diameters were measured by an image-processing technique is represented by the blue dashed line, whereas the computed cumulative polynomial curve is represented by the red line. The mean particle diameters of AgNPs were calculated from SEM by the image-processing technique proposed in this study and then compared with the mean particle size attained by theoretical calculation. The particle-size results can be found in Table 1. These SEM results reveal that the size of AgNps synthesized from a 0.1 mol/L solution concentration lay in the range of 250–700 nm with an average particle size of 392 nm. We observed that the size of AgNps synthesized from 0.2 and 0.3 mol/L lay in the range of 300–800 nm and 400–900 nm, and mean particle size was found to be 503 and 622 nm, respectively, which is comparable with the values listed in Table 1. 

## 4. Conclusions

AgNPs were synthesized using the USP method by varying precursor concentrations. Analysis of the size and size distribution of the synthesized AgNPs was carried out via DLS, theoretical calculation, and an image-processing technique from SEM micrographs. A search algorithm based on the image-processing technique to analyze particle size and size distribution was proposed.

With this proposed new method, the size of AgNP particles on SEM images having a nonhomogeneous background contrast could be measured. Since particles synthesized via the USP method had a pure and spherical shape, we could calculate their diameters through the use of the Hough transform method. Even in the case where a portion of the NPs overlapped with other particles, the diameters of the AgNPs were efficiently calculated. Moreover, our black and white images were further enhanced by the use of an adaptive threshold and subimage creation. 

Thus, the results of measuring AgNP particles on SEM micrographs were computed more reliably by using both the image-processing technique and estimation algorithms. We concluded that the obtained mean particle size from the SEM micrographs was consistent with our theoretical calculation. In addition, NP size distribution observed by our image-processing technique had a broader range than that of DLS analysis. These results show that nanoparticles that cannot be detected by DLS analysis could be measured by the suggested image-processing technique from SEM micrographs.

## Figures and Tables

**Table 1:** Mean particle diameters obtained from theoretical calculation, DLS analysis, and our proposed method.

Sample Codes	Theoretical Mean Particle Diameter	DLS Analysis	Mean Particle Diameter Calculated by Proposed Method
**S1**	410 nm	258 nm	392 nm
**S2**	520 nm	526 nm	503 nm
**S3**	650 nm	653 nm	622 nm
